# Quality evaluation of entrepreneurship education in higher education based on CIPP model and AHP-FCE methods

**DOI:** 10.3389/fpsyg.2022.973511

**Published:** 2022-09-30

**Authors:** Xinqiao Fan, Siyu Tian, Zhenglan Lu, Yirong Cao

**Affiliations:** ^1^Hangzhou Vocational and Technical College, Hangzhou, China; ^2^College of Business, Shanghai University of Finance and Economics, Shanghai, China; ^3^School of Tourism and Urban Rual Planning, Zhejiang Gongshang University, Zhejiang, China; ^4^Hangzhou College of Commerce, Zhejiang Gongshang University, Zhejiang, China

**Keywords:** entrepreneurship education, education evaluation, CIPP model, AHP method, FCE method

## Abstract

Entrepreneurship education has become an important component of higher education development. The purpose of this study is to evaluate entrepreneurship education and determine the extent of satisfaction with the education program. Firstly, based on the CIPP model, this article theoretically analyzes the factors affecting the quality of entrepreneurship education in colleges and universities, and clarifies the keys to improve that education quality. On this basis, using analytic hierarchy process (AHP) and fuzzy comprehensive evaluation (FCE) method, the evaluation index system and fuzzy evaluation model of entrepreneurial education are established. The results show that student participation is the most important factor affecting the quality of entrepreneurship education. Empirical analysis indicates that students have the highest satisfaction with teachers and the lowest satisfaction with the entrepreneurial environment. Apart from convenient and effective measurement of entrepreneurship education, the proposed model provides an important reference for improving the quality of entrepreneurship education in colleges and universities.

## Introduction

In a knowledge-based global market, entrepreneurship is a key factor in enhancing national competitiveness, as it is widely seen as a way to create employment and promote economic growth. It is also an essential means to achieve high levels of competitiveness and innovation in the market. To improve national competitiveness, many countries have embarked on an entrepreneurial revolution over the past two decades (Von Graevenitz et al., 2010; Walter and Block, 2016). For example, there has been a strong momentum in entrepreneurial activity in China over the past decade. The number of enterprises increased rapidly from 9,593,700 in 2011 to more than 30 million in 2021, with an average annual growth rate of more than 21%.^1^ This rapid expansion has been accompanied by similar growth in entrepreneurship education. In the hope of encouraging entrepreneurship, many countries have invested substantial amounts in entrepreneurship education at universities (Von Graevenitz et al., 2010; Kirby et al., 2011; Martin et al., 2013; Lee et al., 2014; Walter and Block, 2016). As early as 1947, Harvard University inaugurated the course “New Business Management,” signaling the beginning of entrepreneurial education in colleges and universities. In 1968, Babson College established a bachelor’s degree in entrepreneurial education, which formed the initial entrepreneurial education system in the United States (Solomon and George, 2007). Early entrepreneurial education in the United States took the “quick success of entrepreneurs” as its main educational concept, aiming to cultivate qualified entrepreneurs, solve the problem of employment, and create the greatest possible benefits for American society (Liñán and Fayolle, 2015). Then, entrepreneurial education was gradually incorporated into general education, with the main goal of cultivating students’ entrepreneurial spirit. Entrepreneurial education not only teaches “how to set up a business” but also imparts the basic knowledge needed in the process of entrepreneurship. It aims to cultivate individuals’ innovation ability, critical thinking and new enterprise management skills (Bechard and Gregoire, 2005). In the early 1980s, entrepreneurship education was introduced to China from the West. After years of development, the concept of entrepreneurship was gradually developed (Xing, 2013). In 2015, The General Office of the State Council in China issued the *Implementation Opinions on Deepening the Reform of Innovation and Entrepreneurship Education in Colleges and Universities*, which raised the development of innovation and entrepreneurship education to a strategic level. The number of colleges and universities offering entrepreneurship-related courses has grown from a few in the 1990s to nearly all institutions.^2^ The worldwide growth and development of courses and programs dedicated to entrepreneurship and innovation has been remarkable in recent years.

A significant amount of literature acknowledges the positive contribution of entrepreneurship education in developing people’s skills and enhancing entrepreneurial attitudes and willingness. Peter Drucker, one of the greatest management thinkers of our time, once said that entrepreneurship is a discipline, and, like any discipline, it can be learned (Drucker, 1985). Most of the empirical studies surveyed indicated that entrepreneurship can be taught, or at least encouraged, by entrepreneurship education (Barba-Sánchez and Atienza-Sahuquillo, 2018; Boldureanu et al., 2020; Cera et al., 2020). Entrepreneurship education is seen as an instrument that fosters entrepreneurship, thereby enhancing one’s capability, skills and motivation to become an entrepreneur (Walter et al., 2013; Shirokova et al., 2018; Barba-Sánchez et al., 2019).

However, in the process of the large-scale expansion of entrepreneurship education, the quality of entrepreneurship education still faces challenges (Hameed and Irfan, 2019). The evaluation of the teaching quality is affected by various fuzzy and uncertain factors (Zhao and Zheng, 2021). Scholars call for a timely evaluation of entrepreneurship education (Eryanto et al., 2019; Suroso et al., 2020). Evaluation is at the center of all improvements, both the quality of education and the effective functioning of the school. Policy makers and researchers have stressed the need to evaluate education, which helps with quality assurance, quality control, quality monitoring and quality development (Eryanto et al., 2019). Although entrepreneurial education has become a widely taught subject, the evaluation of entrepreneurial education has not been researched enough in the academic context.

The goals of our research are threefold: (i) to identify the factors that affect the quality of entrepreneurship education; (ii) to clarify the influence degree of each factor on the quality of entrepreneurship education and construct an evaluation model; and (iii) to evaluate the quality of entrepreneurship education in colleges and universities based on the model. For this purpose, we have designed several research steps. First, based on the CIPP model, this article uses a method of literature analysis and expert opinions to sort out the influencing factors of entrepreneurship education and designed an evaluation system framework of entrepreneurship education for college students. Second, this article adopts the analytic hierarchy process (AHP) to determine the index weight and construct the index model. AHP refers to a decision-making method that decomposes the elements that related to decision-making into levels such as goals, criteria, and schemes, and then conducts qualitative and quantitative analysis on this basis (Saaty, 2003). Third, taking a university in Zhejiang Province of China as an example, this study uses the fuzzy comprehensive evaluation (FCE) method to evaluate the effectiveness of entrepreneurship education (Yu, 2022). FCE method is a comprehensive evaluation method based on fuzzy mathematics, which converts qualitative evaluation into quantitative evaluation according to the membership theory of fuzzy mathematics (Feng and Xu, 1999). Finally, we discuss the main results concerning the evaluation of entrepreneurship education and present our findings.

## Theoretical foundation

### Educational evaluation model

The evaluation system is one of the important factors affecting the development of entrepreneurship education. Foreign scholars have generally evaluated the implementation of entrepreneurship education in universities in terms of the number of entrepreneurship courses, the entrepreneurship rate of college students, and the financing amount of startup enterprises, and they have given attention to the direct and indirect impact of these factors on the economy and society (Liñán et al., 2011; Cunningham et al., 2017; Lebret, 2017; Guerrero et al., 2018). Research concerning the quality evaluation index system for entrepreneurship education in China is still in its early stages. Based on methodological triangulation, Liu et al. (2021) proposed and validated a model for measuring the effectiveness of entrepreneurship education on the three dimensions of entrepreneurial competencies, barriers, and intentions. The findings show that the improvement of participants’ entrepreneurial competencies, the reduction of entrepreneurial barriers, and the change in entrepreneurial intention reflect the effectiveness of entrepreneurial education for college students. Zhu and He (2020) evaluated the process elements and educational effectiveness of innovation and entrepreneurship education through students’ self-cognition and found that government support, entrepreneurial practice, classroom teaching, personal resources and other educational process elements have a significant impact on the effectiveness of innovation and entrepreneurship education. Huang and Huang (2019) constructed a quality evaluation system for innovation and entrepreneurship education in the context of China from the perspectives of students and teachers, development status evaluation, final result evaluation and implementation process evaluation.

### CIPP model

Some evaluation designs and models have been used to evaluate entrepreneurial education projects, programs or work. Among them, the CIPP model is a decision-oriented evaluation model proposed by Stufflebeam in 1983, which includes four elements: C-context, I-input, P-process, and P-product. This model improves the previous educational evaluation model dominated by Taylor’s behavioral goal evaluation. Taylor’s behavior goal evaluation model takes the educational goal as the important basis of the whole teaching evaluation and judges the teaching effect by the degree of completion of the teaching goal, which is result-oriented. The CIPP model holds that the ultimate goal of evaluation is not proof but dynamic improvement. The most important aspect of this model is that it provides a holistic view of every element by evaluating the context, input, process and output from each and every angle (Boldureanu et al., 2020). Recent authors believe that the CIPP model can be effectively applied to education evaluation (Aziz et al., 2018; Eryanto et al., 2019).

#### Context

Context evaluation helps to assess the needs and opportunities within a defined context or environment (Stufflebeam and Shinkfield, 2007). Environmental factors are an important catalyst for the success of sustainable entrepreneurship programs (Toghrāyi et al., 2019; Chege and Wang, 2020). It was recognized that the development of educational programmes could encourage and strengthen entrepreneurship. For example, study by Chege and Wang (2020) analyzed that promoting environmental awareness in college students’ educational plans would induce a greater willingness to start businesses. Many studies have confirmed that entrepreneurial components such as entrepreneurial policies positively influence students’ climate perception of entrepreneurship (Jansen et al., 2015; Belas et al., 2017; Bergmann et al., 2018). Barba-Sánchez et al. (2022) through the survey of a Spanish university, found that both entrepreneurial behavior attitude and perceived behavioral control had significant effects on the entrepreneurial intention in college students, which corroborated a high degree of environmental awareness in attitude towards behaviour construct. Elizabeth (2020), through a survey of 365 samples from Africa, concluded that the entrepreneurial atmosphere has a substantial impact on youth entrepreneurship in colleges and universities.

#### Input

Input refers to the materials, time and human resources spent to carry out effective work. During the input stage of entrepreneurship education, researchers have mainly focused on mentors for entrepreneurship education programs (Eryanto et al., 2019). Teachers have a professional position in the process of entrepreneurship education (Foliard et al., 2018). Ruskovaara and Pihkala (2015) collected relevant data from basic education to high school education, which showed that the entrepreneurial training received by teachers seemed to be the main factor determining the observable entrepreneurial education provided by teachers.

#### Process

Process includes the teaching processes. Teaching methods and disciplined construction are important factors. Through an experimental study on a sample of 308 undergraduate students in Malaysia, Ismail et al. (2018) concluded that different teaching methods used in entrepreneurship education have different effects on individual skill development. Compared with students using student-centered methods, students using teacher-centered methods achieved statistically higher levels of objective and subjective learning outcomes. Professor Kolb (1981) proposed the theory of “experiential learning” and offered insight into the development of learning styles. This theory proposed that entrepreneurial learning should start from experience, raise questions, doubts and reflections, and finally put the theory into practice to improve learning ability (Gemmell and Kolb, 2013; Naufalin et al., 2016). Fan (2018) claimed that entrepreneurial education is not a purely independent discipline but a comprehensive discipline composed of multiple other disciplines, such as education concerning startup management, corporate culture construction, policy response and market analysis.

#### Product

Product focuses on the quality of teaching learning and its usefulness and the potential benefits to society. The product of the entrepreneurship practice program is the resulting skills gained by the entrepreneurship practice education participants, including that each student can create employment, obtain the ability to work independently (or start a business), have the capability to control the decision-making situation, be capable of producing in the field of entrepreneurship, have professional competitiveness, and have an attitude of creativity and innovation, and that students have awareness of the added value of the skills that have been obtained (Patrick et al., 2016; Eryanto et al., 2019).

Generally, CIPP model has several advantages. CIPP model is a development-oriented evaluation model with dynamic characteristics that can help colleges and universities adjust education evaluation indicators in a timely manner according to social needs. Second, the CIPP model can track the whole process of evaluation and provide evaluation feedback to help the evaluation object optimize its behavior. Finally, the evaluation indicators constructed according to the CIPP model include internal and external factors, as well as process and outcome factors. The constructed indicators are both scientific and systematic, which can provide a basis for decision-making (Turmuzi et al., 2022). Therefore, this article adopts the CIPP model as the theoretical basis for the evaluation of entrepreneurship education quality.

### The method of determining the index weight

Scholars have developed a variety of methods to determine the weight of indicators, such as empirical determination method, Delphi method, AHP and entropy weight method (Saaty, 2003; Zou et al., 2006; Hsu et al., 2010). AHP is one of the most used method in recent years. It divides the problems to be solved into multiple different factors and levels according to specific membership relations, and then calculates the importance ranking of the component factors of each level relative to the target element (Saaty, 2003). AHP has the following advantages: First of all, it takes the research object as a system and makes decisions according to the thinking mode of decomposition-comparison-judgment-synthesis. It is an important tool for systematic analysis (de FSM Russo and Camanho, 2015). Secondly, it is a kind of concise and practical method since it combines qualitative with quantitative methods organically (Liu et al., 2020). Thirdly, when quantitative data information is insufficient, AHP can make up for this deficiency since this method mainly starts with the evaluator’s understanding of the essential elements of the evaluation problem, and emphasizes qualitative analysis and judgment more than general quantitative methods (Liu et al., 2020).

### Fuzzy comprehensive evaluation

Fuzzy comprehensive evaluation method usually refers to an evaluation method based on the theory of fuzzy mathematics. It turns qualitative evaluation into quantitative evaluation by the application of membership theory, and makes comprehensive evaluation of related influencing factors based on fuzzy mathematics (Feng and Xu, 1999). Generally speaking, the application of FCE method usually includes the following stages: firstly, the set up the evaluation index; secondly, the expert method, entropy weight method or AHP are selected to obtain the weights of different indicators; thirdly, establish the evaluation matrix; finally, integrate the evaluation matrix and index weight, then select the factor with the largest correlation to implement integration and elaborate the result vector (Chen et al., 2015).

Due to the complexity of evaluation factors and the hierarchy of evaluation objects, there may be fuzzy influencing factors in the proposed evaluation criteria, or the indicators are not easy to quantify. These problems make it impossible to directly choose “yes” or “no” to describe the factors. FCE method selects a number of indicators to analyze the subordination level of the evaluation object, and scientifically plans the change interval of the evaluation object (Feng and Xu, 1999). FCE can realize the full integration of quantitative and qualitative, and improve the credibility of evaluation results (Zhao and Zheng, 2021).

### The application of AHP – FCE

From the application process and connotation of FCE model, it can be seen that it mainly depends on the hierarchical analysis model to determine the relevant indicators. Because of its theoretical basis and strong practicability, AHP-FCE method has been widely applied in the field of education in recent years. Yang et al. (2019) evaluated the simulation teaching quality of *Fundamental Nursing Curriculum* with FCE method, and provided a scientific evaluation method for the improvement of simulation teaching. Zhao and Zheng (2021) used AHP, fuzzy system theory and grey theory to probe deep into the fuzzy evaluation of physical education in colleges. The proposed fuzzy evaluation model serves an innovative tool to evaluate the quality of college physical education. In Chen’s study (Chen et al., 2015), the AHP-FCE approach is used to evaluate the standard of teaching quality. AHP-FCE has been proved to be a scientific and feasible methodology in education (Chen et al., 2015; Yang et al., 2019).

## Study design

### Method

Educational evaluation is a process of value judgment with respect to educational activities, processes and results, and it can provide a reference standard for future educational activities. Such evaluation can not only be used to measure the quality of the educational process and its results but also plays a certain guiding role in the whole educational teaching process. The evaluation of education quality involves many aspects, such as course setting, teacher team construction, and social support. This evaluation has many characteristics, such as multiple attributes, multiple levels and fuzziness, so quantitative analysis is difficult. In this article, the AHP and FCE methods are combined to construct an evaluation index system for entrepreneurship education quality, and the quality of entrepreneurship education in colleges and universities is evaluated and analyzed.

### Analytic hierarchy process

The AHP is a kind of decision-making method combining qualitative and quantitative analysis that can evaluate multiple indices and multiple levels and has the advantages of featuring simple and clear calculations and a high systematic value (Vaidya and Kumar, 2006). The AHP is generally combined with the Delphi method to determine the weight of indicators. The steps involved in this process are to construct a hierarchy model and determine its membership. The model includes the target layer (A layer), the main criterion layer (B layer), the criterion layer (C layer) and the index layer. Second, through expert consultation and scoring, the two factors are compared in each element of the B and C layers, and the judgment matrix is constructed. The establishment of the expert evaluation table is grounded on the basic principle of the nine-point scale method. The expert determines the relative importance and the degree of superiority and inferiority of the two factors in the index comparison and fills in the appropriate importance degree. The consistency index is C_I_ = $\frac{\lambda_{\text{max}}-n}{n-1}$ , where, $\lambda_{\text{max}}$ represents the maximum characteristic root of the matrix and n is the order of the matrix. At the same time, it is also necessary to determine whether the random consistency ratio (*C*_R_ = $\frac{C_{I}}{R_{I}}$ ) is less than 0.1, which means that consistency ratio verification is successful. If the results show that the consistency test is not satisfied, experts’ opinions should be sought, and the scoring results should be adjusted appropriately until the consistency test is satisfied and the weight should be calculated. Finally, the total order of hierarchy and its consistency test are calculated.

### Fuzzy comprehensive evaluation

Fuzzy comprehensive evaluation is based on fuzzy mathematics, which can express the fuzzy relationships among various factors by quantitative and accurate mathematical methods. To solve the fuzzy problem with respect to the evaluation of various factors, FCE produces clear and systematic results (Jin et al., 2004; Pittaway and Cope, 2007). The steps are to first determine the factor set F and evaluation set E. Factor set *F* = {*f_i_*}, *i* = 1, 2, …, *n*; evaluation set *E* = {*e_j_*} = {very satisfactory, satisfactory, generally unsatisfactory, very unsatisfactory}, and the measurement scale vectors *H* = [5, 4, 3, 2, 1]. Then, the single factor evaluation fuzzy membership vector *R_i_ =* (*r*_*i*1_, *r*_*i*2_, *r*_in_), *i* = 1, 2, …, *n*, forms the membership vector *R* = (*R*_1_, *R*_2_, *R_n_*)*^T^* = (*R_ij_*). Second, the first-level FCE is conducted, the weight matrix W_F_ is determined, and the comprehensive evaluation vector, that is, the fuzzy membership matrix and weight matrix contained in the criterion layer B = W_F_R, is constructed. Finally, the second-level FCE is carried out, and the final judgment result S = W_2_ M is calculated, where W_2_ is the weight matrix of each index in criterion layer B relative to the target layer and M is the fuzzy evaluation matrix composed of the first-level comprehensive evaluation results of each index in criterion layer B.

### Index selection

Combining the CIPP model with existing research, this study intends to analyze the quality of entrepreneurship education from four aspects: entrepreneurial environment, faculty, course setting, and student participation. Among these aspects, the entrepreneurial environment represents the atmosphere of the school’s entrepreneurship education, the faculty represents the school’s input in entrepreneurship education, the course setting refers to the process form of the school’s entrepreneurship education, and the participation of students reflects the output of entrepreneurship education. We also conducted 12 in-depth qualitative studies to determine the evaluation metrics (Barba-Sánchez et al., 2019). We interviewed people from 4 groups: leaders of entrepreneurship education in colleges and universities, teachers of entrepreneurship education in colleges and universities, government departments in charge of entrepreneurship education, and business entrepreneurship mentors. After integrating expert opinions, the target layer of the evaluation system of entrepreneurship education in colleges and universities is set as the evaluation system of entrepreneurship education for college students. The criterion layer includes four parts: entrepreneurial environment, faculty, course setting and student participation. The scheme layer is the specific index of each criterion layer (see Table 1). Taking into account the effects of courses, teachers, university environment and students’ own characteristics on the results of entrepreneurship education, the first-level indicators are established based on these four elements. Based on the establishment of first-level indicators, a total of 16 second-level indicators reflecting the effect of entrepreneurship education are selected (see Table 1).

**Table 1:** Evaluation criteria of the entrepreneurship education quality evaluation system.

Criterion layer (first-level indicators)	Model	Evaluation index	Evaluation criteria
B1 entrepreneurial environment	Context	C1 number of entrepreneurial clubs	The number of student organizations such as entrepreneurial societies
		C2 number of seminars held	The number of entrepreneurship-related forum activities
		C3 number of entrepreneurship competitions held	The number of entrepreneurial competitions
		C4 student resource coverage	The coverage rate of students supported by funds, venues and policies
B2 faculty	Input	C5 academic background	The lecturers have a good academic background
		C6 entrepreneurial experience	The lecturers have entrepreneurial experience
		C7 management ability	The lecturer used to be an enterprise management talent and has rich practical experience
		C8 scientific research capability	The lecturer has research-related entrepreneurial achievements, such as having published articles, presided over projects, etc.
B3 course setting	Process	C9 theory teaching	Entrepreneurship courses help students improve and accumulate the knowledge structure of entrepreneurship
		C10 practice teaching	Entrepreneurship courses can improve students’ practical abilities
		C11 teaching methods	Teaching methods can stimulate students’ enthusiasm for learning and increase participation
		C12 degree of integration with major	Entrepreneurship education courses can be combined with major-field courses to improve professional ability
B4 student participation	Product	C13 number of students with work experience	Number of students with work, internship or part-time experience
		C14 number of innovation achievements	Innovation achievements include technology, management, product and other innovation achievements
		C15 number of activities attended	The number of students participating in entrepreneurial activities, scientific research activities, club activities and social activities
		C16 practical ability	The amount of time and energy students invest in entrepreneurship and the number of practical activities they participate

## Analytic hierarchy process analysis

This study designed a questionnaire according to Table 1, and invited experts from relevant departments, universities and enterprises to make suggestions on the indicators of the questionnaire. In addition, experts were invited to score the relative importance of each indicator by using the 1–9 scale method. According to Okoli and Pawlowski (2004), it would be practical to solicit 10 to 18 members in size. A total of 15 valid questionnaires were received, including 3 managers from government departments, 9 principals and teachers from colleges and universities, and 3 mentors from enterprise. Yaahp software was used for weight calculation and consistency test of AHP.

### Consistency test of first-level indicators

The consistency test is conducted for the quality evaluation of entrepreneurship education for college students at the target level. The first-level indicators include four elements: entrepreneurial environment (B1), faculty (B2), course setting (B3), and student participation (B4). The judgment matrix A of the first-level index layer about the target layer is as follows:


$$A=\left[\begin{array}{ccccc} A & B1 & B2 & B3 & B4\\ B1 & 1 & 0.65 & 3.7 & 1.24\\ B2 & 1.53 & 1 & 2 & 0.86\\ B3 & 0.27 & 0.5 & 1 & 1.24\\ B4 & 0.8 & 1.16 & 2.43 & 1 \end{array}\right]$$

The calculated C_R_ = 0.0418 < 0.1 indicates that the consistency of matrix A is good and within the acceptable range.

### Consistency test of secondary indicators

The second-level indicators corresponding to the entrepreneurial environment (B1) include the number of associations (C1), number of seminars held (C2), number of entrepreneurship competitions held (C3), and coverage rate of funded students (C4). The judgment matrix B1 of the first-level index layer pertaining to the target layer is clarified, and the C_R_ of matrix B1 is 0.07.

The second-level indicators involved in faculty (B2) include four indicators: academic background (C5), entrepreneurial experience (C6), entrepreneurial management ability (C7), and scientific research ability (C8). The judgment matrix B2 is constructed, and the C_R_ of matrix B2 is 0.01, less than 0.1.

There are four second-level indicators corresponding to course theory teaching (C9), course practice teaching (C10), teaching methods (C11), and teaching quality (C12) in the course setting (B3). The hierarchical analysis model and the entrepreneurial education quality evaluation matrix B3 are established. The C_R_ of matrix B3 is 0.07, indicating that the consistency of matrix B3 is within the acceptable range.

The second-level indicators of student participation (B4) are the number of students with work experience (C13), number of entrepreneurial achievements (C14), number of activities attended (C15) and practical ability (C16). The AHP model and evaluation matrix B4 are established. The C_R_ of matrix B4 is 0.03.

The calculation matrices B1, B2, B3 and B4 of the second-level indicators are as follows:


$$\begin{array}{c} B1=\left[\begin{array}{ccccc} B1 & C1 & C2 & C3 & C4\\ C1 & 1 & 1.53 & 0.6 & 0.35\\ C2 & 0.65 & 1 & 0.75 & 1\\ C3 & 1.66 & 1.33 & 1 & 0.75\\ C4 & 2.85 & 1 & 1.33 & 1 \end{array}\right]\\ B2=\left[\begin{array}{ccccc} B2 & C5 & C6 & C7 & C8\\ C5 & 1 & 0.28 & 0.42 & 0.73\\ C6 & 3.57 & 1 & 1.5 & 2.7\\ C7 & 2.38 & 0.66 & 1 & 2.8\\ C8 & 1.36 & 0.37 & 0.35 & 1 \end{array}\right] \end{array}$$

$$\begin{array}{c} B3=\left[\begin{array}{ccccc} B3 & C9 & C10 & C11 & C12\\ C9 & 1 & 0.3 & 0.58 & 0.43\\ C10 & 3.3 & 1 & 7.6 & 2.1\\ C11 & 1.72 & 0.13 & 1 & 0.73\\ C12 & 2.32 & 0.47 & 1.36 & 1 \end{array}\right]\\ B4=\left[\begin{array}{ccccc} B4 & C13 & C14 & C15 & C16\\ C13 & 1 & 1.76 & 2.6 & 1.5\\ C14 & 0.56 & 1 & 3 & 1.4\\ C15 & 0.38 & 0.33 & 1 & 3.39\\ C16 & 0.66 & 0.71 & 0.29 & 1 \end{array}\right] \end{array}$$

The judgment matrix is constructed for the evaluation model *via* the AHP, and the consistency ratios of the constructed judgment matrix are all less than 0.1, indicating that the consistency of the matrix is good and that the evaluation model constructed is reasonable.

### Determination of the weight of single-level sorting

After analysis of all the matrices, the index weights of the evaluation hierarchy of college students’ entrepreneurship education are summarized, as shown in the table. Students and the environment have a great impact on the evaluation of entrepreneurship education, with students accounting for 33.89% of the total weight, the environment accounting for 33.01%, teachers accounting for 22.36% and courses accounting for 9.74% (Table 2).

**Table 2:** Weight and evaluation of indicators.

Criterion layer (first-level indicators)	Weight	Index (second-level indicators)	Weight	Very dissatisfied	Dissatisfied	Generally	Satisfied	Very satisfied
B1 entrepreneurial environment	0.3301	Number of entrepreneurial clubs	00.1424	6	36	110	146	199
		Number of seminars held	00.2251	8	68	140	94	187
		Number of entrepreneurship competitions held	00.2493	7	57	152	90	191
		Student resource coverage	00.3832	8	24	156	124	185
B2 faculty	0.2336	Academic background	00.1036	16	24	78	158	221
		Entrepreneurial experience	00.3322	12	34	94	146	211
		Management ability	00.3784	13	22	59	178	225
		Scientific research capability	00.1858	10	48	108	138	193
B3 course setting	0.0974	Theory teaching	00.1016	22	44	134	120	177
		Practice teaching	00.3860	14	18	152	132	181
		Teaching methods	00.2115	16	30	96	150	205
		Degree of integration with major	00.3008	15	23	106	154	199
B4 student participation	0.3389	Number of students with work experience	0.3333	11	27	122	152	187
		Number of innovation achievements	0.2896	8	50	106	150	183
		Number of activities attended	0.2478	8	42	104	150	193
		Practical ability	0.1293	21	45	134	120	177

## Fuzzy mathematical evaluation of entrepreneurship education

### Data collection

The Entrepreneurship Education Evaluation Form was designed for college students to investigate students’ satisfaction with courses, teachers and other aspects. This study selected a college in Zhejiang Province of China as the research object. The college has been offering entrepreneurship education to all students since 2016. At present, entrepreneurship education has covered all majors. The school has also been listed as a pilot school for entrepreneurship education in Zhejiang Province. In order to ensure the validity of the evaluation, students who have received or are receiving entrepreneurship education courses are selected as the research objects. According to the school’s official data, the total number of students in this academic year was 6,185, of which 3,046 had received entrepreneurship education. The research started in December 2019 and lasted until March 2020. A total of 297 valid questionnaires were collected. The evaluation results of each index were obtained through statistical collation of the questionnaire, as shown in Table 2.

### Fuzzy comprehensive evaluation of indexes

Fuzzy evaluation matrices R1, R2, R3, and R4 are obtained by the fuzzy statistical method as follows:


$$\begin{array}{c} R1=|\begin{array}{ccccc} 0.0121 & 0.0724 & 0.2213 & 0.2938 & 0.4004\\ 0.0161 & 0.1368 & 0.2817 & 0.1891 & 0.3763\\ 0.0161 & 0.1127 & 0.3058 & 0.1811 & 0.3843\\ 0.0161 & 0.0483 & 0.3139 & 0.2495 & 0.3722 \end{array}|\\ R2=|\begin{array}{ccccc} 0.0322 & 0.0483 & 0.1569 & 0.3179 & 0.4447\\ 0.0241 & 0.0684 & 0.1891 & 0.2938 & 0.4245\\ 0.0201 & 0.0443 & 0.1247 & 0.3581 & 0.4527\\ 0.0201 & 0.0966 & 0.2173 & 0.2777 & 0.3883 \end{array}|\text{.} \end{array}$$

$$\begin{array}{c} R3=|\begin{array}{ccccc} 0.0443 & 0.0885 & 0.2696 & 0.2414 & 0.3561\\ 0.0282 & 0.0362 & 0.3058 & 0.2656 & 0.3642\\ 0.0322 & 0.0604 & 0.1932 & 0.3018 & 0.4125\\ 0.0322 & 0.0443 & 0.2133 & 0.3099 & 0.4004 \end{array}|\\ R4=|\begin{array}{ccccc} 0.0201 & 0.0523 & 0.2455 & 0.3058 & 0.3763\\ 0.0201 & 0.0523 & 0.2777 & 0.2736 & 0.3763\\ 0.0161 & 0.1006 & 0.2133 & 0.3018 & 0.3682\\ 0.0161 & 0.0845 & 0.2093 & 0.3018 & 0.3883 \end{array}|\text{.} \end{array}$$

Then, the first-level fuzzy evaluation is carried out to construct criterion layer B, and the weighted average M (⋅, +) operator is used as the fuzzy product operation of the weight coefficient matrix W and evaluation matrix R to obtain the comprehensive evaluation vector of B1.

Similarly, the comprehensive evaluation vectors of B2, B3, B4, B5, and B6 can be obtained:


$$\begin{array}{c} B1=|0.1424\;0.2251\;0.2493\;0.3832|\\ \;|\begin{array}{ccccc} 0.0121 & 0.0724 & 0.2213 & 0.2938 & 0.4004\\ 0.0161 & 0.1368 & 0.2817 & 0.1891 & 0.3763\\ 0.0161 & 0.1127 & 0.3058 & 0.1811 & 0.3843\\ 0.0161 & 0.0483 & 0.3139 & 0.2495 & 0.3722 \end{array}|\\ =|\begin{array}{ccccc} 0.0155 & 0.0877 & 0.2915 & 0.2252 & 0.3802 \end{array}|\text{.} \end{array}$$

$$\begin{array}{c} B2=|\begin{array}{cccc} 0.1036 & 0.3322 & 0.3784 & 0.1858 \end{array}|\\ \;|\begin{array}{ccccc} 0.0322 & 0.0483 & 0.1569 & 0.3179 & 0.4447\\ 0.0241 & 0.0684 & 0.1891 & 0.2938 & 0.4245\\ 0.0201 & 0.0443 & 0.1247 & 0.3581 & 0.4527\\ 0.0201 & 0.0966 & 0.2173 & 0.2777 & 0.3883 \end{array}|\\ =|\begin{array}{ccccc} 0.0227 & 0.0624 & 0.1667 & 0.3176 & 0.4306 \end{array}|\text{.} \end{array}$$

$$\begin{array}{c} B3=|\begin{array}{cccc} 0.2115 & 0.3860 & 0.1016 & 0.3008 \end{array}|\\ \;|\begin{array}{ccccc} 0.0322 & 0.0604 & 0.1932 & 0.3018 & 0.4125\\ 0.0282 & 0.0362 & 0.3058 & 0.2656 & 0.3642\\ 0.0443 & 0.0885 & 0.2696 & 0.2414 & 0.3561\\ 0.0322 & 0.0443 & 0.2133 & 0.3099 & 0.4004 \end{array}|\\ =|\begin{array}{ccccc} 0.0319 & 0.0491 & 0.2505 & 0.2841 & 0.3845 \end{array}| \end{array}$$

$$\begin{array}{c} B4=|\begin{array}{cccc} 0.3234 & 0.2868 & 0.1328 & 0.2570 \end{array}|\\ \;|\begin{array}{ccccc} 0.0201 & 0.0523 & 0.2455 & 0.3058 & 0.3763\\ 0.0201 & 0.0523 & 0.2777 & 0.2736 & 0.3763\\ 0.0161 & 0.1006 & 0.2133 & 0.3018 & 0.3682\\ 0.0161 & 0.0845 & 0.2093 & 0.3018 & 0.3883 \end{array}|\\ =|\begin{array}{ccccc} 0.0186 & 0.0670 & 0.2411 & 0.2950 & 0.3783 \end{array}|\text{.} \end{array}$$

Finally, the fuzzy comprehensive evaluation matrix of the criterion layer is as follows:


$$R_{B}=|\begin{array}{c} B1\\ B2\\ B3\\ B4 \end{array}|=|\begin{array}{ccccc} 0.0155 & 0.0877 & 0.2915 & 0.2252 & 0.3802\\ 0.0227 & 0.0624 & 0.1667 & 0.3176 & 0.4306\\ 0.0319 & 0.0491 & 0.2505 & 0.2841 & 0.3845\\ 0.0186 & 0.0670 & 0.2411 & 0.2950 & 0.3783 \end{array}|$$

Then, the second-level FCE is carried out, and the fuzzy evaluation of R_B_ is carried out with M (⋅, +).

Then, we obtain the comprehensive evaluation vector of the school’s entrepreneurship education with $S={M_{B}}^{*}R_{B}|\begin{array}{ccccc} 0.0198 & 0.0710 & 0.2413 & 0.2762 & 0.3917 \end{array}|$ .

Based on the above calculation results, the evaluation results of entrepreneurship education quality are shown in Table 3.

**Table 3:** Fuzzy comprehensive evaluation results of entrepreneurship education quality.

Index	Assessment results				
	Very dissatisfied	Dissatisfied	Generally	Satisfied	Very satisfied
B1 entrepreneurial environment	0.0155	0.0877	0.2915	0.2252	0.3802
B2 faculty	0.0227	0.0624	0.1667	0.3176	0.4306
B3 course setting	0.0319	0.0491	0.2505	0.2841	0.3845
B4 student participation	0.0186	0.0670	0.2411	0.2950	0.3783
Comprehensive assessment	0.0198	0.0710	0.2413	0.2762	0.3917

Table 3 shows that the membership degree value of entrepreneurship education quality is 0.3917, which is in the very satisfied range according to the principle of maximum membership degree. According to Table 3, the evaluation results are further converted into component values, and the evaluation set is B = {very dissatisfied, dissatisfied, average, satisfied, very satisfied} = {0, 25, 50, 75, 100}. The score and order of each indicator are shown in Table 4. Table 4 shows that the faculty ranks first, indicating that interviewees have a high recognition of the entrepreneurship guidance teacher team.

**Table 4:** Index scores and ranking results.

Index	Score	Rank
B1 entrepreneurial environment	71.6681	4
B2 faculty	76.7731	1
B3 course setting	73.5067	3
B4 student participation	73.6878	2

## Discussion of results

Entrepreneurship education is becoming increasingly critical, as it can lead to positive outcomes such as the activation of innovative thinking and improvement of employability. Based on the CIPP program, this study constructed a quality evaluation system for entrepreneurship education, including the entrepreneurial environment, faculty, course setting and student participation. The quality of entrepreneurship education in colleges and universities was comprehensively evaluated by AHP and FCE. The order of importance of each evaluation index in the criterion layer is student participation > entrepreneurial environment > faculty > course setting. The index of “student participation” has the greatest influence on the quality evaluation of entrepreneurship education, accounting for 33.89% of the total effect. In the satisfaction evaluation, faculty scored 76.7731 points, ranking first. This indicates that teachers and students are satisfied with the entrepreneurship education teacher team. Ranking fourth is the entrepreneurship environment, which means that the entrepreneurship atmosphere in colleges and universities is not strong enough. According to the above results, this study puts forward the following suggestions.

First, entrepreneurship education should attach importance to the subject of entrepreneurship education. In the second-level indicator of “students,” students with work experience have the greatest influence, accounting for 11.11% of the total. Improving the quality of talent training is the foundation of running a school. Therefore, during training, the subject status of students should be highlighted, and a teaching method that features equal interaction between teachers and students should be actively constructed (Fayolle, 2018). In terms of talent training, the three-level interaction model of “campus + community + enterprise” is constructed to promote cooperation and training to create abundant internship and practice opportunities for students.

The second factor that needs our attention is the creation of an entrepreneurial environment in colleges and universities. The index of “environment” also has a great influence on the quality evaluation of entrepreneurship education, accounting for 33.01% of the total effect. In the implementation of entrepreneurship education, we can increase the number of entrepreneurship associations, entrepreneurship competitions and seminars to create a good atmosphere (Naufalin et al., 2016).

In addition, a contingent of high-quality teachers should be developed. Teachers have a professional position in the process of entrepreneurship education (Foliard et al., 2018). Unlike the traditional education model, entrepreneurship education emphasizes not only theoretical knowledge but also practical experience. Therefore, in the input stage of entrepreneurship education, we should pay attention to the construction of the team of mentors (Eryanto et al., 2019). At present, most colleges and universities draw teachers from other majors to teach entrepreneurship courses, and their theoretical knowledge and practical ability cannot meet the needs of entrepreneurial education. To promote the in-depth and lasting development of entrepreneurship education, the teacher team should be composed of full-time teachers, on-campus professional teachers and off-campus entrepreneurship mentors. Full-time or part-time training helps teachers accumulate management experience, improve their practical abilities, and improve the quality of entrepreneurship education.

The establishment of the course system also requires attention. Different teaching methods in entrepreneurship education have different effects on individual skill development (Ismail et al., 2018). In the quality evaluation of entrepreneurship education in colleges and universities, the “course setting” plays a decisive role. The establishment of the course system should focus on cultivating students’ theoretical knowledge and practical abilities (Gemmell et al., 2013). Measures such as strengthening practical ability training and improving the level of professional core courses should be adopted to promote the reform and exploration of the entrepreneurship education courses setting system.

## Implications

In terms of theoretical implications, first, this research improves the entrepreneurship education quality evaluation system and enriches the theory of education evaluation. On the basis of relevant studies on education evaluation theory, this study takes universities in Zhejiang Province of China as the research object and CIPP education evaluation theory as the basis and then takes the entrepreneurship environment, teachers, teaching system and student participation as the basic content to build a framework model for entrepreneurship education quality evaluation. This is a response to Aziz et al. (2018) and other scholars’ calls for further development of educational evaluation theory.

Second, this study expands the research method of entrepreneurial education quality evaluation. Previous scholars have mostly evaluated the quality of entrepreneurial education at the theoretical level, while empirical studies are few (Eryanto et al., 2019). Scholars call for enriching the quantitative analysis methods of educational quality evaluation to improve the scientificity and feasibility of educational quality evaluation (Turmuzi et al., 2022). This study adopted the AHP method to construct an index system and used FCE for verification and analysis. The analysis methods used in this study provide an empirical reference for subsequent scholars to evaluate the quality of entrepreneurship education in colleges and universities.

In terms of practical significance, this study conducts research on the construction and application of an entrepreneurship education evaluation system and proposes countermeasures to improve the quality development of entrepreneurship education in local colleges and universities through demonstration and extraction. Education authorities can draw on the research results to formulate and adjust the development planning of entrepreneurship education. For example, the survey results show that the entrepreneurial environment has the lowest evaluation score. Schools should strengthen entrepreneurial support and enrich entrepreneurial activities to create a strong entrepreneurial atmosphere and further stimulate students’ willingness to innovate and start businesses.

## Limitation and future directions

This study has two main limitations. Firstly, the selection of quality evaluation indicators of entrepreneurship education may not be comprehensive enough. The construction of the index system of this study mainly refers to relevant policy data and literature. The index design only selects course setting, entrepreneurship environment, teaching staff and student participation, while other aspects, for example, the subject of enterprises, is not involved in the evaluation. The subjects involved in the evaluation indicators are not limited to universities and students, and employers and governments can also be included in the future research. Secondly, in the application research of evaluation system, considering the accuracy of the data collection, this study only selected the universities in Zhejiang province as sample schools. Due to the shortage of sample range and capacity, the results may have some deviation. In the future research, universities of different levels and categories can be selected for empirical analysis, so that the evaluation system will be more convincing.

## Data availability statement

The raw data supporting the conclusions of this article will be made available by the authors, without undue reservation.

## Author contributions

XF: conceptualization, methodology, investigation, and writing – original draft. ST: data collection and writing – original draft. ZL: data collection and data analysis. All authors contributed to the article and approved the submitted version.

## Funding

The study received funding from Vocational Education Research Project (ZJCV2021C10), Zhejiang Province, China and Hangzhou Philosophy and Social Science Research Base (Hangzhou Industry-education Integration Research Institute) Project (2021).

## Conflict of interest

The authors declare that the research was conducted in the absence of any commercial or financial relationships that could be construed as a potential conflict of interest.

## Publisher’s note

All claims expressed in this article are solely those of the authors and do not necessarily represent those of their affiliated organizations, or those of the publisher, the editors and the reviewers. Any product that may be evaluated in this article, or claim that may be made by its manufacturer, is not guaranteed or endorsed by the publisher.

## Footnotes

^1^Source: National Bureau of Statistics, *China statistical yearbook*: http://www.stats.gov.cn/tjsj./ndsj/

^2^Source: The State Council, The People’s Republic Of China, *Implementation Opinions of The General Office of the State Council on Deepening the Reform of Innovation and Entrepreneurship Education in Institutions of Higher Learning*: http://www.gov.cn/zhengce/content/2015-05/13/content_9740.htm
